# NT-A9, a new triazole, exhibits potent antifungal activity against *Candida* and *Cryptococcus* species through potent ergosterol biosynthesis inhibition

**DOI:** 10.1128/aac.00768-26

**Published:** 2026-08-05

**Authors:** Shuo Zhu, Tingjunhong Ni, Lu Gao, Wanqian Li, Dazhi Zhang, Wenjuan Wu, Yuanying Jiang, Hui Lu

**Affiliations:** 1 Department of Pharmacy, Shanghai Tenth People’s Hospital, School of Medicine, Tongji University 12476, Shanghai, China; 2 School of Pharmacy, Naval Medical University 12521 , Shanghai, China; 3 Department of Laboratory Medicine, Shanghai East Hospital, School of Medicine, Tongji University 12476, Shanghai, China; 4 Key Laboratory of Pathogen-Host Interaction, Ministry of Education, School of Medicine, Tongji University 12476, Shanghai, China; University of Iowa, Iowa City, lowa, USA

**Keywords:** NT-a9, triazoles, Erg11, ergosterol biosynthesis, antifungal resistance, invasive fungal infections

## Abstract

Invasive fungal infections (IFIs) remain a major global health threat due to limited therapeutic options and rising azole resistance. NT-a9 is a novel triazole antifungal with potent activity against *Candida* and *Cryptococcus* species via strong inhibition of ergosterol biosynthesis. *In vitro*, NT-a9 exhibited potent and sustained antifungal activity against clinical isolates, with superior potency compared to fluconazole (FLC). It also displayed a prolonged post-antifungal effect (PAFE) against most *Candida* species, a property rarely seen in conventional azoles. Resistance profiling demonstrated that NT-a9 possesses a high genetic barrier, fully suppressing resistant mutant emergence in *Cryptococcus* species and maintaining stable susceptibility in *Candida albicans* during 28 days of continuous exposure. NT-a9 retained potent activity against FLC-resistant *Cryptococcus neoformans* and multidrug-resistant *Candida auris*, with minimal influence from drug efflux mechanisms. In murine invasive candidiasis, a single low dose of NT-a9 achieved 100% survival and markedly reduced renal fungal burden, significantly outperforming FLC. NT-a9 also substantially improved survival in mice infected with FLC-resistant *C. auris*. Mechanistically, NT-a9 binds Erg11 with much higher affinity than FLC by forming key hydrogen bonds with Gly307 and His377, leading to irreversible ergosterol depletion, toxic sterol accumulation, and severe fungal membrane damage. Transcriptomic analysis further confirmed that NT-a9 extensively disrupts sterol biosynthesis and triggers compensatory gene expression that reinforces its antifungal action. As a novel triazole integrating long-lasting efficacy, a high resistance barrier, and potent activity against pan-azole-resistant pathogens, NT-a9 represents a promising clinical candidate for treating IFIs.

## INTRODUCTION

Invasive fungal infections (IFIs) have become an increasingly critical global health challenge, driven by the growing prevalence of immunocompromised individuals and the widespread application of modern medical interventions (1). These infections are associated with an estimated 6.5 million new cases and 3.8 million deaths each year, and *Candida*, *Cryptococcus*, and *Aspergillus* species together account for more than 85% of life-threatening IFIs (2). The continuous emergence of antifungal resistance, particularly azole resistance, has further worsened clinical outcomes and severely limited treatment options for hospitalized patients (3, 4).

Currently, only three major classes of antifungal agents are available for clinical use: polyenes, echinocandins, and azoles (5). Among these, polyenes are limited by severe and dose-dependent nephrotoxicity (6), whereas echinocandins are restricted by a narrow antifungal spectrum and high economic cost (7). Azoles remain the cornerstone of IFI therapy due to their convenient oral administration, broad-spectrum activity, and generally acceptable safety profiles (8). However, currently approved azole drugs display substantial limitations that compromise their long-term clinical utility. Fluconazole (FLC), the most widely prescribed azole, has been widely used for the treatment of *Candida* and *Cryptococcus* infections; however, its efficacy is increasingly undermined by the development of fungal resistance and tolerance (3, 4). Although voriconazole (VRC), another typical azole drug, has good anti-*Aspergillus fumigatus* activity (9), it is not a first-line drug for treating infections caused by *Candida albicans* and *Cryptococcus neoformans* (10). Additionally, the clinical use of VRC is limited by poor pharmacokinetic properties, significant hepatotoxicity, nephrotoxicity, and the necessity for real-time therapeutic monitoring (11–13). Most conventional azoles also lack a prolonged post-antifungal effect (PAFE), resulting in short-lived activity and the need for frequent dosing (14–16). Furthermore, clinically used azoles typically present a low genetic barrier to resistance, allowing resistant mutants to emerge rapidly during prolonged therapy (17–19). In addition, few available azoles exhibit reliable efficacy against multidrug-resistant *Candida auris*, an emerging pathogen that poses a severe global threat (20–22). Together, these critical drawbacks highlight the urgent need for novel triazole derivatives with improved potency, sustained action, a higher resistance barrier, and activity against resistant pathogenic fungi.

**Fig 1:**
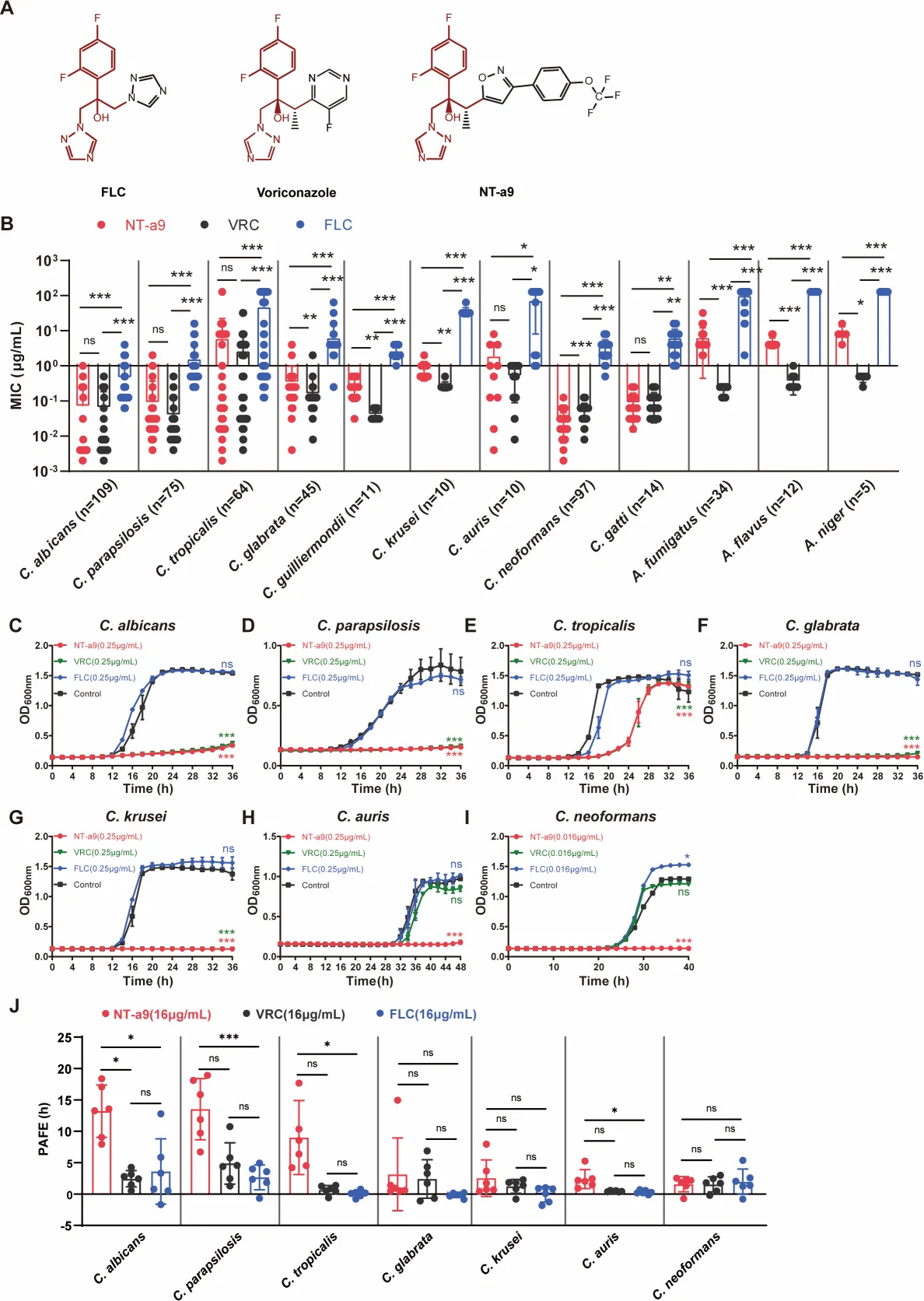
NT-a9 demonstrates potent, prolonged, and sustained antifungal activity. (**A**) The chemical structure of fluconazole (FLC), voriconazole (VRC), and NT-a9. (**B**) MIC of NT-a9, VRC, and FLC against *Candida*, *Cryptococcus*, and *Aspergillus* species. (**C–I**) Time-growth curves of *C. albicans*, *C. parapsilosis*, *C. tropicalis*, *C. glabrata*, *C. krusei*, *C. auris*, and *C. neoformans*, treated with or without NT-a9, VRC, and FLC at the same concentration. (**J**) The PAFE of *C. albicans*, *C. parapsilosis*, *C. tropicalis*, *C. glabrata*, *C. krusei*, *C. auris*, and *C. neoformans*. NT-a9, VRC, and FLC at 16 μg/mL were co-cultured with fungal strains for 4 h. The time-growth curves of tested strains were dynamically monitored every 15 min. The PAFE was calculated from the time-growth curves after the drugs were withdrawn. Statistical significance was calculated using one-way ANOVA (ns no significance, *P < 0.05, **P < 0.01, ***P < 0.001).

In our previous studies, we designed and synthesized NT-a9 (Fig. 1A), a novel triazole compound, and demonstrated that it possesses a longer *in vivo* half-life, wider therapeutic window, and potent antifungal activity against *C. neoformans* (23, 24). In the present study, we comprehensively evaluate the antifungal properties of NT-a9 in comparison with FLC against a broad range of clinical *Candida* and *Cryptococcus* isolates. We systematically investigate its *in vitro* potency, sustained antifungal activity, post-antifungal effect, and resistance potential, including mutation prevention and long-term resistance evolution. We also assess the *in vivo* efficacy of NT-a9 in murine models of invasive candidiasis and FLC-resistant *C. auris* infection. Finally, we elucidate the detailed molecular mechanism by which NT-a9 binds to Erg11 with high affinity and robustly inhibits ergosterol biosynthesis. Collectively, our findings demonstrate that NT-a9 overcomes key limitations of current azoles by displaying potent and sustained activity, a high genetic barrier to resistance, and excellent efficacy against drug-resistant pathogens, supporting its further development as a promising clinical candidate for the treatment of refractory IFIs.

## RESULTS

### NT-a9 has a broad antifungal spectrum and exhibits potent and sustainable antifungal activity

To characterize the antifungal spectrum and potency of NT-a9, we tested a large collection of clinical fungal isolates obtained from multiple hospitals, including *C. albicans* (*n* = 109), *Candida parapsilosis* (*n* = 75), *Candida tropicalis* (*n* = 64), *Candida glabrata* (*n* = 45), *Candida guilliermondii* (*n* = 11), *Candida krusei* (*n* = 10), *C. auris* (*n* = 10), *C. neoformans* (*n* = 97), *Cryptococcus gattii* (*n* = 14), *A. fumigatus* (*n* = 34), *Aspergillus flavus* (*n* = 12), and *Aspergillus niger* (*n* = 5). Antifungal susceptibility testing demonstrated that NT-a9 exhibited *in vitro* antifungal potency comparable to voriconazole (VRC) and markedly stronger than FLC across all tested fungal pathogens (Fig. 1B and Table 1). NT-a9 exerted broad-spectrum inhibitory activity against *Candida* spp., *Cryptococcus* spp., and *Aspergillus* spp. For NT-a9, the MIC range was 0.004–8 μg/mL, with MIC₉₀ values ranging from 0.063 to 16 μg/mL, geometric mean (GM)-MIC values from 0.011 to 8.000 μg/mL, and a modal MIC of 0.004–8 μg/mL across all tested isolates. VRC displayed similarly potent antifungal activity with narrow MIC fluctuations, whereas FLC yielded substantially higher MIC values, especially against azole-resistant strains. Notably, NT-a9 showed prominent inhibitory advantages against azole-resistant isolates, including *C. krusei* and *C. auris*, against which FLC exhibited very weak antifungal activity. Species-specific MIC₅₀, MIC₉₀, GM-MIC, and modal MIC data for the three agents are summarized in Table 1 and visualized in Fig. 1.

**TABLE 1:** MIC range, MIC90, MIC50, modal MIC, and GM-MIC of NT-a9, voriconazole, and fluconazole^*a*^

Species	Number	NT-a9						VRC					FLC			
		MIC range	MIC_90_	MIC_50_	Modal MIC	GM- MIC	MIC range	MIC_90_	MIC_50_	Modal MIC	GM- MIC	MIC range	MIC_90_	MIC_50_	Modal MIC	GM-MIC
*C. albicans*	*n* = 108	0.002– 1	0.25	0.004	0.004	0.011	0.002–0.5	0.25	0.008	0.004	0.013	0.063–4	1	0.125	0.125	0.236
*C. parapsilosis*	*n* = 75	0.004– 2	0.125	0.031	0.031	0.030	0.004–0.5	0.125	0.016	0.008	0.018	0.25–16	2	0.5	0.5	0.758
*C. tropicalis*	*n* = 64	0.002–128	16	0.125	8	0.388	0.004–32	4	0.125	0.031	0.285	0.125–128	128	1	128	5.301
*C. glabrata*	*n* = 45	0.004– 4	0.5	0.25	0.25	0.193	0.008– 2	0.25	0.125	0.125	0.107	0.25–64	8	4	4	4.189
*C. guilliermondii*	*n* = 11	0.031–0.5	0.5	0.25	0.25	0.207	0.031–0.063	0.063	0.031	0.125	0.040	1–4	4	2	2	2.416
*C. krusei*	*n* = 10	0.5–2	1	0.5	0.5	0.758	0.25– 0.5	0.25	0.25	0.25	0.268	32–64	32	32	32	34.297
*C. auris*	*n* = 10	0.004 –8	4	0.5	0.125	0.381	0.008–1	1	0.5	1	0.250	1–128	128	64	128	19.698
*C. neoformans*	*n* = 97	0.002–0.125	0.063	0.016	0.031	0.020	0.008–0.125	0.125	0.063	0.063	0.051	0.5–8	8	4	4	3.084
*C. gatti*	*n* = 14	0.016–0.25	0.25	0.063	0.031	0.066	0.031–0.25	0.25	0.063	0.031	0.076	1–16	16	4	4	4.416
*A. fumigatus*	*n* = 34	2–32	16	4	4	5.006	0.125–0.25	0.25	0.25	0.25	0.200	2–128	128	128	128	76.889
*A. flavus*	*n* = 12	4–8	8	4	4	4.490	0.25–0.5	0.5	0.25	0.25	0.334	128–128	128	128	128	128.000
*A. niger*	*n* = 5	4–16	16	8	8	8.000	0.25–0.5	0.5	0.5	0.5	0.435	128–128	128	128	128	128.000

^*a*^ Values of ≤0.002 μg/mL are treated as 0.002 μg/mL, and values of >64 μg/mL are treated as 128 μg/mL. GM-MIC, Geometric Mean of Minimum Inhibitory Concentration.

For subsequent experiments, we selected seven representative species, including *C. albicans, C. parapsilosis, C. tropicalis, C. glabrata, C. krusei, C. auris*, and *C. neoformans*, with three strains per species. Time-growth curves were generated to evaluate the real-time dynamic antifungal activity of NT-a9, VRC, and FLC at equivalent concentrations, a more informative measure of antifungal efficacy than single-point MIC determination. FLC exerted minimal inhibitory effects against all tested strains at the concentrations used (Fig. 1C through J). In contrast, NT-a9 maintained strong antifungal activity against *C. albicans* (Fig. 1C), *C. parapsilosis* (Fig. 1D), *C. glabrata* (Fig. 1F), and *C. krusei* (Fig. 1G) for at least 36 h and against rapidly growing *C. tropicalis* for 24 h (Fig. 1E), with potency comparable to VRC. Notably, NT-a9 produced sustained suppression of *C. auris* for at least 48 h, whereas FLC and VRC showed no meaningful activity relative to the untreated control (Fig. 1H). Similarly, NT-a9 at 0.016 μg/mL exerted durable growth inhibition against *C. neoformans* for up to 40 h (Fig. 1I).

We further evaluated the persistence of antifungal activity following drug removal by determining the PAFE, a key pharmacodynamic parameter for identifying long-acting antifungal candidates. Although NT-a9 showed only a short PAFE against *C. neoformans* (1.560 ± 1.206 h) (Fig. 1J), it displayed significantly prolonged PAFE values against *C. albicans* (13.210 ± 4.171 h), *C. parapsilosis* (13.530 ± 4.882 h), *C. tropicalis* (9.002 ± 5.886 h), and *C. auris* (2.382 ± 1.501 h). These values were all significantly higher than those of FLC (3.594 ± 5.199 h, 2.662 ± 1.982 h, 0.034 ± 0.526 h, and 0.296 ± 0.300 h, respectively; *P* < 0.05 or *P* < 0.001) (Fig. 1J). Moreover, the PAFE of NT-a9 against *C. albicans* was significantly longer than that of VRC (13.210 ± 4.171 h vs 2.436 ± 1.299 h). The PAFE values of NT-a9 against *C. parapsilosis, C. tropicalis*, and *C. auris* were also numerically higher than those of VRC, although the differences did not reach statistical significance (Fig. 1J).

Collectively, these results demonstrate that NT-a9 displays potent and sustained antifungal activity against *C. albicans, C. parapsilosis, C. tropicalis, C. glabrata, C. krusei, C. auris*, and *C. neoformans*, exceeding that of FLC and comparable to that of VRC. Importantly, NT-a9 retains durable residual antifungal activity after drug removal in most *Candida* species.

### *C. albicans* and *Cryptococcus* species exhibit a low propensity to develop resistance to NT-a9

Despite the potent *in vitro* antifungal activity of NT-a9, the long-term clinical utility of azoles is often limited by the emergence of drug resistance. We therefore comprehensively evaluated the resistance potential of NT-a9 in parallel with FLC and VRC. The mutation prevention concentration (MPC), defined as the lowest drug concentration that completely blocks the growth of resistant mutants, was determined by plating high-inoculum fungal suspensions (2 × 10⁶ CFUs per plate) on agar containing serial dilutions of each agent, followed by 5 days of incubation.

**Fig 2:**
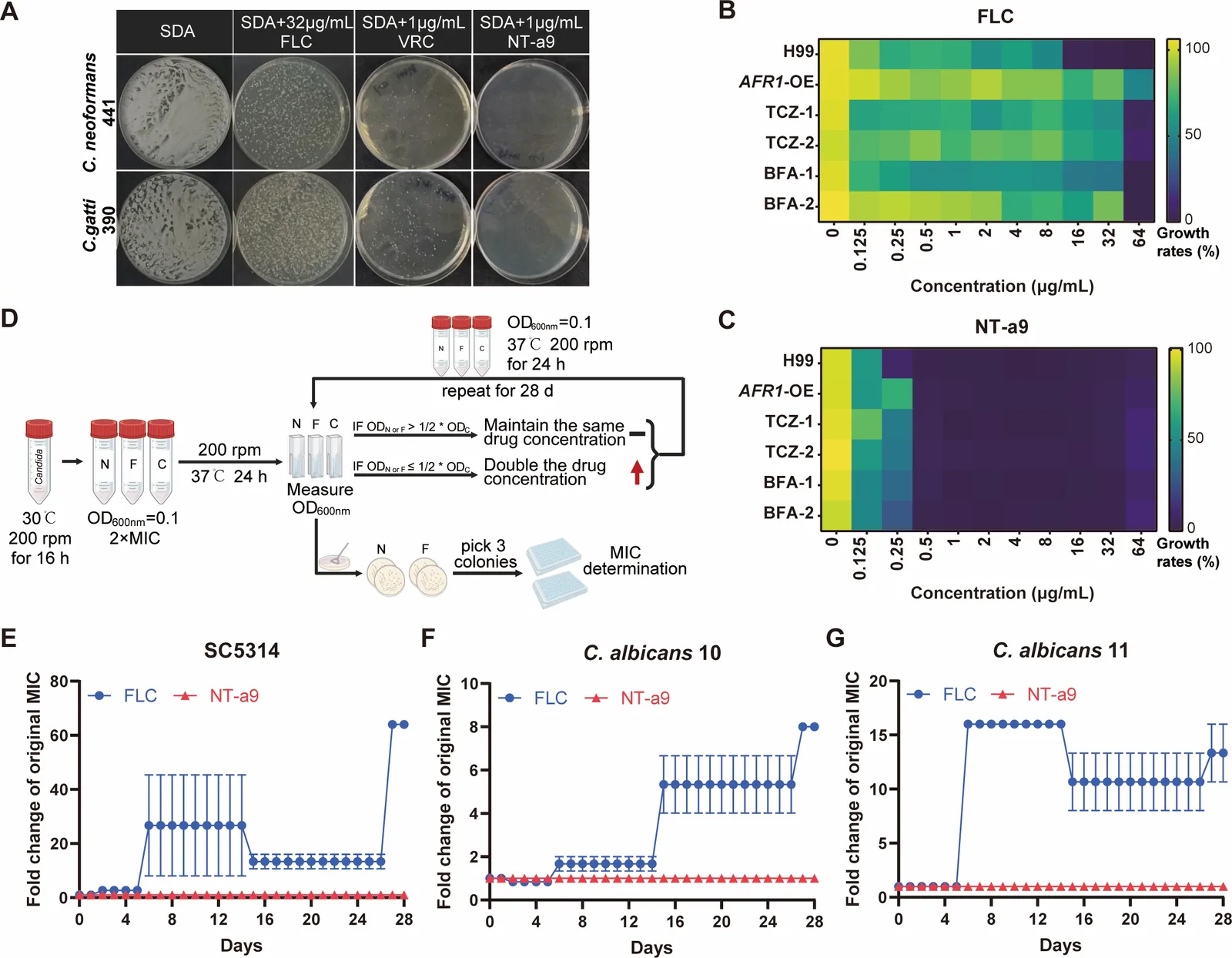
NT-a9 does not readily induce the production of resistant mutants. (**A**) The display of MPC results on SDA plates. (**B and C**) MIC of FLC or NT-a9 against *C. neoformans,* involving H99, *AFR1*-OE strain, TCZ-1, TCZ-2, BFA-1, and BFA-2. (**D**) The procedure of *in vitro* experimental resistance evolution under long-term drug exposure. (**E–G**) The resistance development of standard *C. albicans* SC5314, clinical *C. albicans* 10, and clinical *C. albicans* 11.

High MPC values were observed for NT-a9, VRC, and FLC against most *Candida* species (Table 2). In contrast, while FLC showed a very high MPC (64 μg/mL) against *C. neoformans*, NT-a9 and VRC displayed exceptionally low MPC values of 0.5 μg/mL and 1 μg/mL, respectively, indicating superior ability to prevent resistance emergence (Table 2). We extended this analysis to additional *C. neoformans* isolates and *C. gattii*. Abundant resistant colonies appeared on FLC-containing plates at 32 μg/mL, and sporadic colonies were observed on VRC-containing plates at 0.5 μg/mL, whereas 0.5 μg/mL NT-a9 fully suppressed the emergence of resistant mutants (Fig. 2A). These results confirm that NT-a9 strongly prevents resistance development in both *C. neoformans* and *C. gattii*, with activity slightly superior to that of VRC (Table 2).

**TABLE 2:** The MPC values for NT-a9, VRC, and FLC against Candida and *Cryptococcus species*

Species	Isolate	MPC (μg/mL)		
		NT-a9	VRC	FLC
*C. albicans*	21	>128	>128	>128
	22	>128	>128	>128
	25	>128	>128	>128
*C. tropicalis*	172	>128	>128	>128
	177	>128	>128	>128
	182	>128	>128	>128
*C. parapsilosis*	104	>128	>128	>128
	105	32	32	>128
	106	>128	>128	>128
*C. glabrata*	202	>128	>128	>128
	203	>128	>128	>128
	204	>128	>128	>128
*C. krusei*	471	128	32	>128
	472	128	32	>128
	474	128	32	>128
*C. auris*	276	>128	>128	>128
	277	>128	>128	>128
	278	>128	>128	>128
*C. neoformans*	431	1	2	>32
	432	0.5	1	>32
	433	1	2	>32
	434	0.5	1	>32
	435	0.5	2	>32
	436	0.5	1	>32
	437	0.5	2	>32
	438	0.5	1	>32
	439	1	2	>32
	440	0.5	1	>32
	441	1	2	>32
	442	1	2	>32
	443	0.5	1	64
	444	128	128	>128
	445	1	2	>128
*C. gattii*	386	2	2	>32
	387	1	2	>32
	388	1	2	>32
	389	1	2	>32
	390	1	2	>32
	392	1	2	>32
	393	1	2	>32
	394	1	2	>32
	395	1	2	>32
	396	1	2	>32
	397	1	1	>32
	399	2	2	>32

Drug efflux mediated by ABC transporters represents a major azole resistance mechanism. We therefore tested NT-a9 activity against a *C. neoformans* strain overexpressing the efflux pump gene *AFR1* (AFR1-OE). For the parental strain H99, the MIC of FLC was 16 μg/mL, while that of NT-a9 was 0.25 μg/mL (Fig. 2B and C). In the AFR1-OE strain, the MIC of FLC increased to >64 µg/mL, whereas the MIC of NT-a9 rose only modestly to 0.5 μg/mL, indicating that drug efflux has minimal impact on NT-a9 activity (Fig. 2B and C). We further tested four FLC-resistant *C. neoformans* derivatives (TCZ-1, TCZ-2, BFA-1, and BFA-2) with induced aneuploidy. The MIC of NT-a9 remained unchanged at 0.25 μg/mL against all four strains relative to the parental H99 (Fig. 2B and C), demonstrating no cross-resistance between NT-a9 and FLC in *C. neoformans*.

To assess resistance development under long-term drug exposure, we performed a 28-day serial passage experiment with *C. albicans* in the presence of NT-a9 or FLC at 2× MIC (Fig. 2D). Under continuous FLC pressure, MIC values increased dramatically by 64-fold, 8-fold, and 13-fold for the reference strain SC5314 and clinical isolates 10 and 11, respectively, indicating substantial resistance development (Fig. 2E through G). In striking contrast, the MICs of NT-a9 remained stable throughout the 28-day passage period in all tested *C. albicans* strains (Fig. 2E through G), indicating an extremely high genetic barrier to resistance.

Collectively, these findings demonstrate that both *C. albicans* and *Cryptococcus* species display a very low propensity to develop resistance to NT-a9, supporting its potential for sustained clinical use.

### NT-a9 demonstrates potent *in vivo* efficacy against murine invasive candidiasis

Building on the potent and sustained *in vitro* activity of NT-a9, we further evaluated its *in vivo* efficacy using a murine model of invasive candidiasis. Mice were infected via tail-vein injection with *C. albicans* SC5314 cells (2 × 10⁶ CFU per mouse) and treated 24 h later with a single oral dose of NT-a9 (1 mg/kg or 0.25 mg/kg) or FLC (2 mg/kg or 0.5 mg/kg); control mice received vehicle only (Fig. S1A). The MIC values of NT-a9 and FLC against *C. albicans* SC5314 were 0.016 μg/mL and 0.125 μg/mL, respectively.

**Fig 3:**
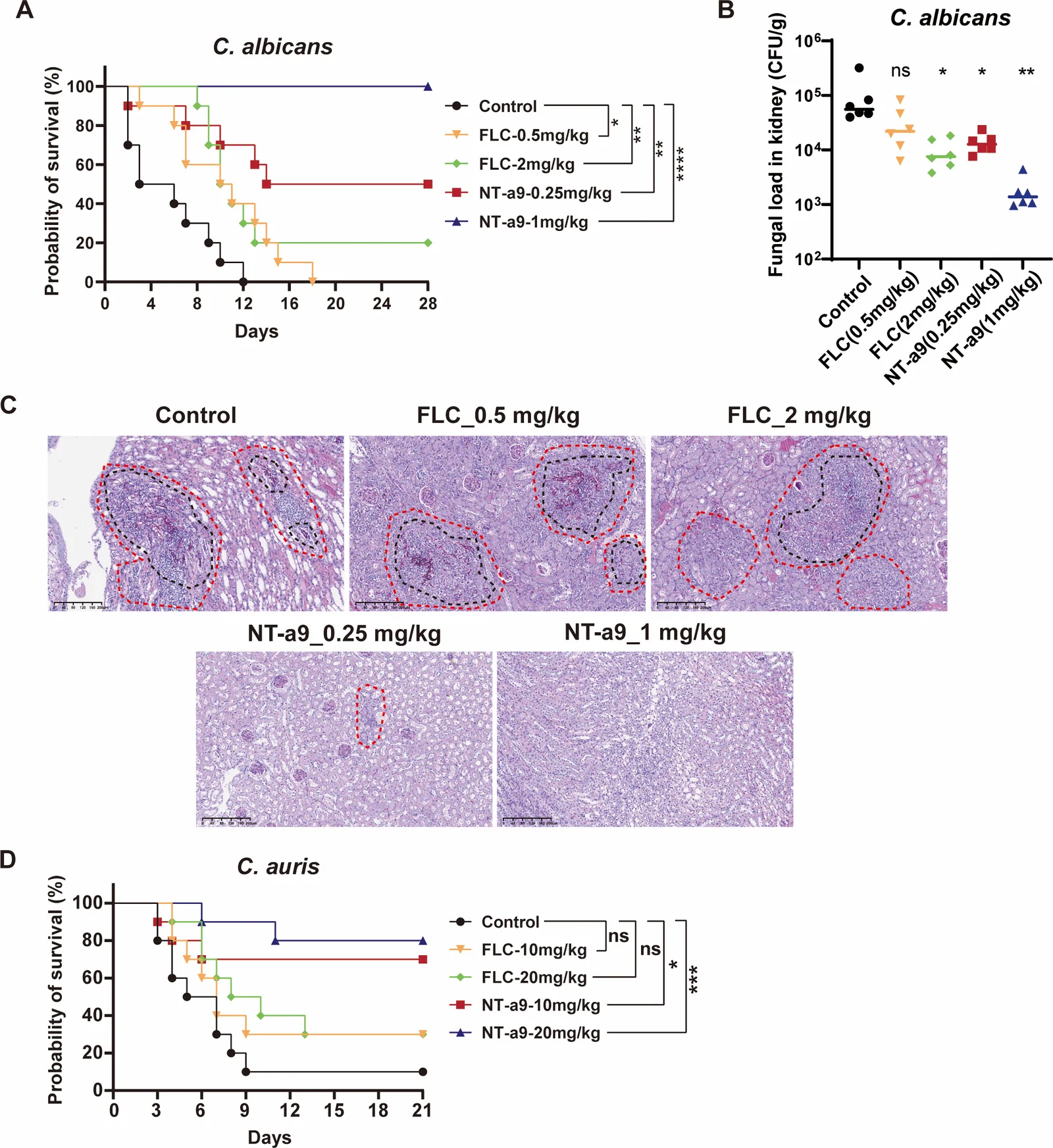
Antifungal efficacy of NT-a9 against invasive fungal infections in a mouse model. (**A**) Survival of infected mice under drug treatment. *C. albicans* SC5314 (2 × 10⁶ cells/mouse) was injected into ICR mice via the tail vein on Day 0. At 24 h post-infection, the drug (NT-a9 or FLC) was administered by oral gavage (single treatment). Mouse survival was monitored daily for 28 days. Statistical significance was calculated using log-rank test (*P < 0.1, **P < 0.01, ***P < 0.001, ****P < 0.0001). (**B**) Renal fungal load of infected mice under drug treatment. On Day 3 post-infection, the right kidneys of infected mice were collected for renal fungal load quantification. Statistical significance was calculated using one-way ANOVA (*P < 0.05, **P < 0.01, ***P < 0.001). (**C**) PAS staining in the kidney of infected mice under drug treatment. On Day 3 post-infection, the left kidneys of infected mice were collected for histopathological analysis. The black dots represent the *C. albicans* invasion, and the red squares represent the inflammatory cell infiltration. (**D**) Therapeutic efficacy of NT-a9 against systemic *C. auris* infection. Survival curves compare NT-a9 (10, 20 mg/kg) versus FLC (10, 20 mg/kg) and vehicle control in immunocompromised mice (*n* = 10/group). Treatments were administered orally at 4 h post-infection with 2 × 10⁷ CFU *C. auris* 278. Statistical significance was calculated using log-rank test (ns no significance, *P < 0.1, **P < 0.01, ***P < 0.001, ****P < 0.0001).

Over a 28-day observation period, untreated mice had a median survival of 12 days (Fig. 3A). Treatment with 0.5 mg/kg FLC prolonged median survival to 18 days (*P* < 0.01), whereas 2 mg/kg FLC increased the survival rate to 20% (*P* < 0.001). Notably, 0.25 mg/kg NT-a9 increased survival to 50% (*P* < 0.001), and 1 mg/kg NT-a9 provided full protection with 100% survival (*P* < 0.0001) (Fig. 3A).

At 3 days post-infection, renal fungal burden and histopathology were analyzed (Fig. S1A). NT-a9 at 0.25 mg/kg reduced renal fungal load to a level comparable to that achieved with 2 mg/kg FLC (Fig. 3B). Treatment with 1 mg/kg NT-a9 led to a striking reduction in fungal burden from 1.002 ± 1.088 × 10⁶ CFU/g to 1.819 ± 1.315 × 10³ CFU/g (*P* < 0.01) (Fig. 3B). Periodic acid-Schiff (PAS) staining showed extensive *C. albicans* invasion and inflammatory infiltration in kidneys from untreated and 0.5 mg/kg FLC-treated mice, while 2 mg/kg FLC partially reduced *C. albicans* colonization (Fig. 3C). Mice receiving 0.25 mg/kg NT-a9 showed no detectable *C. albicans* cells in renal tissue, despite mild residual inflammation. Remarkably, 1 mg/kg NT-a9 completely cleared renal infection, with no observable *C. albicans* cells or inflammation (Fig. 3C). These results confirm that NT-a9 provides superior *in vivo* protection against invasive *C. albicans* infection.

We next evaluated the efficacy of NT-a9 against invasive candidiasis caused by *C. auris*, an emerging multidrug-resistant pathogen of global concern (25, 26). Due to its relatively low virulence, a murine immunosuppression model was used (27). Mice were rendered immunocompromised with cyclophosphamide (200 mg/kg intraperitoneally) 72 h prior to infection and then challenged intravenously with a FLC-resistant clinical *C. auris* isolate (strain 278; 2 × 10⁷ CFU per mouse). Treatment was initiated 4 h post-infection by oral administration of NT-a9 or FLC (Fig. S1B). The MIC values of NT-a9 and FLC against *C. auris* 278 were 1 μg/mL and >64 µg/mL, respectively.

Survival was monitored for 21 days. Untreated control mice exhibited 90% mortality by day 9, with only 10% survival (Fig. 3D). Treatment with FLC at 10 mg/kg or 20 mg/kg provided only modest benefit, increasing survival to 30% without statistical significance. In contrast, NT-a9 showed dose-dependent efficacy, protecting 70% of mice at 10 mg/kg (*P* < 0.01) and 80% at 20 mg/kg (*P* < 0.001) (Fig. 3D). These data demonstrate that NT-a9 exerts robust therapeutic efficacy against invasive candidiasis caused by FLC-resistant *C. auris*.

### NT-a9 potently inhibits ergosterol biosynthesis in *C. albicans*

**Fig 4:**
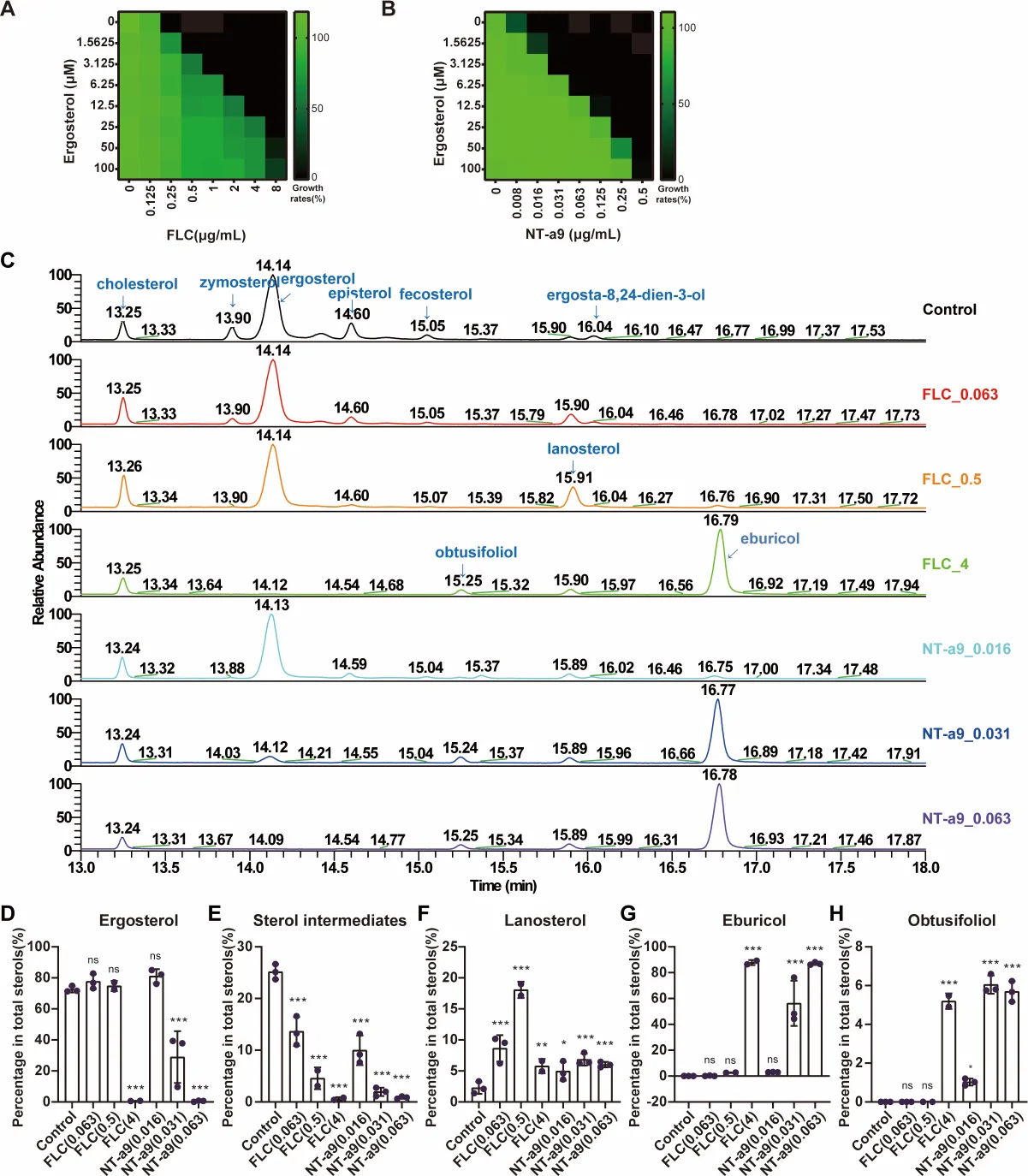
NT-a9 demonstrates high binding affinity for *C. albicans* Erg11. (**A and B**) Fungal growth of *C. albicans* SC5314 in the presence of FLC or NT-a9 combined with varying concentrations of ergosterol, as assessed by checkerboard MIC assays. Fungal growth rates are represented as heatmaps. (**C**) Gas chromatogram of sterols in *C. albicans* under drug treatment. *C. albicans* SC5314 was co-cultured with NT-a9 or FLC at various concentrations for 12 h to extract cell membrane sterol components. The sterol components were analyzed using GC-MS, with cholesterol added as an internal reference during the sterol extraction process. The sterol components were identified based on MS data. Detected sterols included cholesterol, zymosterol, ergosterol, episterol, fecosterol, ergosta-8,24-dien-3-ol, lanosterol, obtusifoliol, and eburicol. (**D–H**) Percentage of ergosterol, sterol intermediates, lanosterol, eburicol, and obtusifoliol in all sterol contents.

NT-a9 shares a similar core structure with classical azoles such as FLC (Fig. 1A). As azole antifungals act primarily by inhibiting lanosterol 14α-demethylase (encoded by the *ERG11 gene in C. albicans*) to block ergosterol biosynthesis, we hypothesized that NT-a9 acts as a triazole analog that competitively inhibits Erg11 and disrupts ergosterol production. In support of this idea, supplementation with exogenous ergosterol significantly restored the growth of *C. albicans* SC5314 during NT-a9 treatment (Fig. 4A and B), confirming that ergosterol inhibition is central to the antifungal mechanism of NT-a9.

To further verify this mechanism, we extracted total sterols from *C. albicans* after 12 h of treatment with graded concentrations of NT-a9 or FLC, using an untreated group as a control. Sterol composition was quantified by gas chromatography-mass spectrometry (GC-MS) with cholesterol as an internal standard (Fig. 4C). In untreated cells, ergosterol accounted for 72.57% ± 2.00% of total sterols, with sterol intermediates comprising 25.16 ± 1.48% (Fig. 4D and E).

Treatment with FLC at 0.063 μg/mL or 0.5 μg/mL inhibited the formation of sterol intermediates and caused lanosterol accumulation but did not reduce ergosterol levels or induce the accumulation of alternative pathway sterols (eburicol or obtusifoliol) (Fig. 4D through H). In contrast, at a high concentration (4 μg/mL), FLC drastically reduced ergosterol to 0.62% ± 0.11% (*P* < 0.001), while eburicol and obtusifoliol increased to 87.81% ± 1.96% (*P* < 0.001) and 5.20% ± 0.40% (*P* < 0.001), respectively (Fig. 4D and G and H).

Strikingly, NT-a9 at a low concentration (0.031 μg/mL) strongly inhibited ergosterol synthesis (28.91% ± 16.71%, *P* < 0.001) and induced marked accumulation of eburicol (56.25% ± 17.48%, *P* < 0.001) and obtusifoliol (6.05% ± 0.47%, *P* < 0.001) (Fig. 4D and G and H). Together, these results demonstrate that NT-a9 inhibits ergosterol biosynthesis with significantly greater potency than FLC in *C. albicans*.

### NT-a9 induces profound membrane damage in *C. albicans*

**Fig 5:**
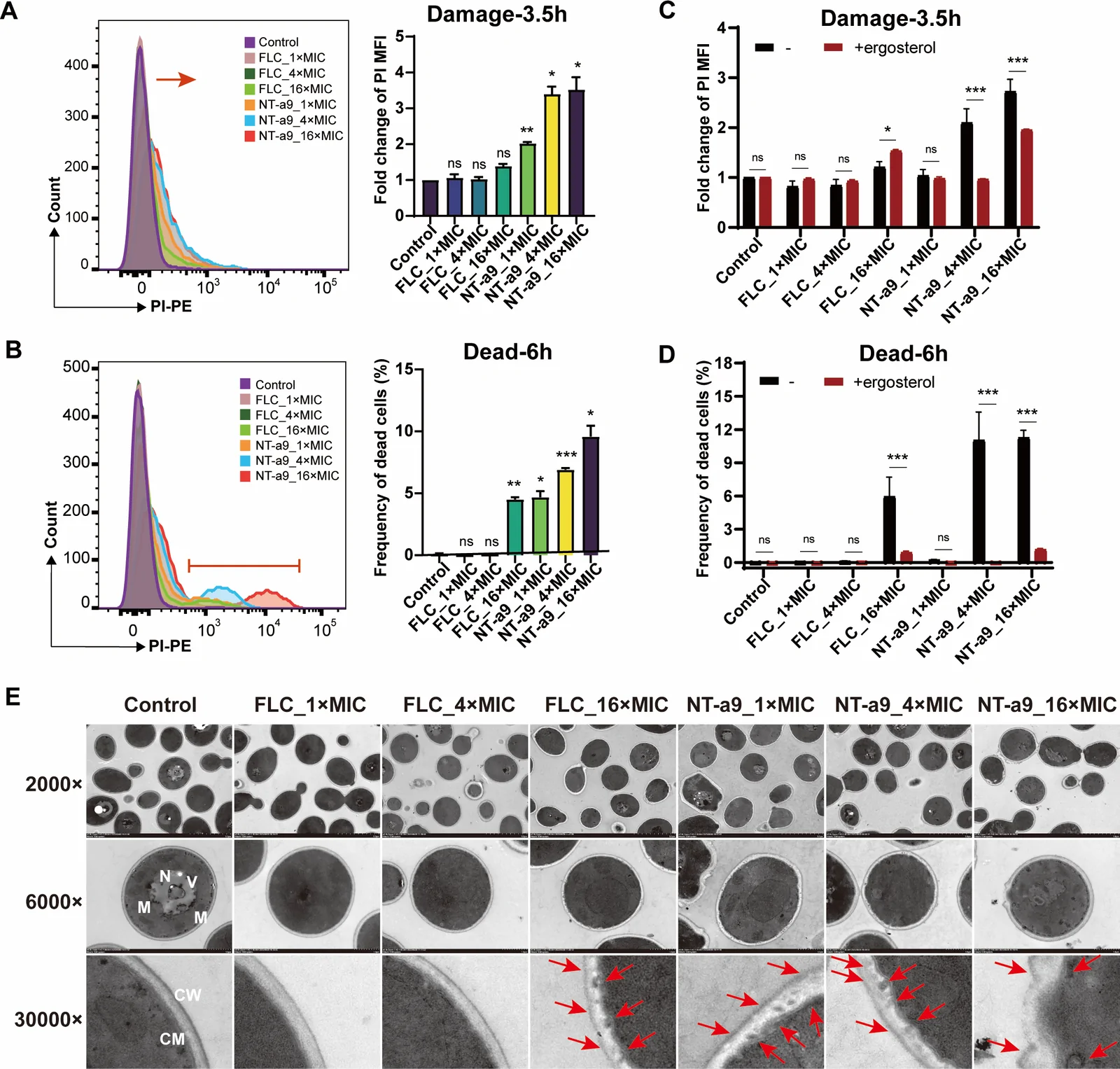
NT-a9 induces cell death by inhibiting ergosterol biosynthesis. (**A**) *C. albicans* SC5314 was co-cultured with the drug for 3.5 h and the PI staining was performed for 30 min. The red arrows on the left indicate the rightward shift of the peaks, while the right graph shows the ratio of the MFI of PI in each group relative to the control group. (**B**) *C. albicans* SC5314 was co-cultured with the drug for 6 h and then PI staining was performed for 30 min. The red line segment in the left graph marks the range of PI-positive cells (dead cells), and the right graph quantifies the percentage of dead cells. Statistical significance was calculated using one-way ANOVA (ns, no significance; *P < 0.05, **P < 0.01, ***P < 0.001). (**C**) Cell membrane damage in each group at 3.5 h after exogenous ergosterol supplementation. (**D**) Percentage of dead cells in each group at 6 h after exogenous ergosterol supplementation. Statistical significance was calculated using two-way ANOVA (ns, no significance; *P < 0.05, **P < 0.01, ***P < 0.001). (**E**) *C. albicans* SC5314 (2 × 10^6^ cells/mL) was co-cultured with or without NT-a9 or FLC at concentrations of 1×, 4×, and 16× MIC, respectively, for 6 h. Fungal cells were collected for transmission electron microscopy. Red arrows represent abnormal cell membrane and cell wall structures. CW, cell wall; CM, cell membrane; V, vesicle; N, nucleus; M, mitochondria.

We next used propidium iodide (PI) staining to assess cell membrane damage resulting from ergosterol depletion (28). Flow cytometric analysis of *C. albicans* showed that treatment with NT-a9 at 1× MIC, 4× MIC, and 16× MIC, as well as FLC at 16× MIC, for 3.5 h caused a marked rightward shift in PI fluorescence intensity, with significantly higher median fluorescence intensity (MFI) in NT-a9-treated cells, indicating severe membrane disruption (Fig. 5A). After 6 h of incubation, a distinct PI-positive peak appeared, signifying the presence of non-viable cells rather than merely membrane-compromised cells (Fig. 5B). The proportion of dead cells was markedly increased following treatment with NT-a9 at 1× MIC, 4× MIC, and 16× MIC, as well as FLC at 16× MIC. Notably, FLC at 16× MIC achieved only comparable killing to NT-a9 at 1× MIC, confirming the superior antifungal activity of NT-a9 (Fig. 5B). Supplementation with exogenous ergosterol significantly alleviated membrane damage and cell death (Fig. 5C and D), verifying that ergosterol depletion is the primary cause of NT-a9-induced cellular damage.

Transmission electron microscopy (TEM) was performed to directly visualize morphological and ultrastructural damage in *C. albicans* SC5314 after 6 h of treatment with NT-a9 or FLC (Fig. 5E). Untreated cells displayed a typical oval morphology with intact, smooth cell walls and membranes (Fig. 5E). *C. albicans* cells treated with 1× MIC FLC showed no obvious ultrastructural abnormalities, while those exposed to 4× MIC FLC exhibited mild membrane deformation. At 16× MIC, FLC caused reduced membrane electron density, accumulation of toxic sterol intermediates adjacent to the membrane, and membrane invagination. In contrast, NT-a9 at 1× MIC, 4× MIC, and 16× MIC induced far more severe morphological and ultrastructural damage than FLC at 16× MIC (Fig. 5E). These results confirm that NT-a9 disrupts fungal cell membranes much more profoundly than FLC, consistent with the flow cytometric findings.

Collectively, these data demonstrate that NT-a9 strongly inhibits ergosterol biosynthesis, triggers extensive membrane damage and structural failure, and ultimately leads to rapid *C. albicans* cell death-with potency markedly superior to that of FLC.

### NT-a9 binds Erg11 with high affinity to drive superior inhibition of ergosterol biosynthesis

**Fig 6:**
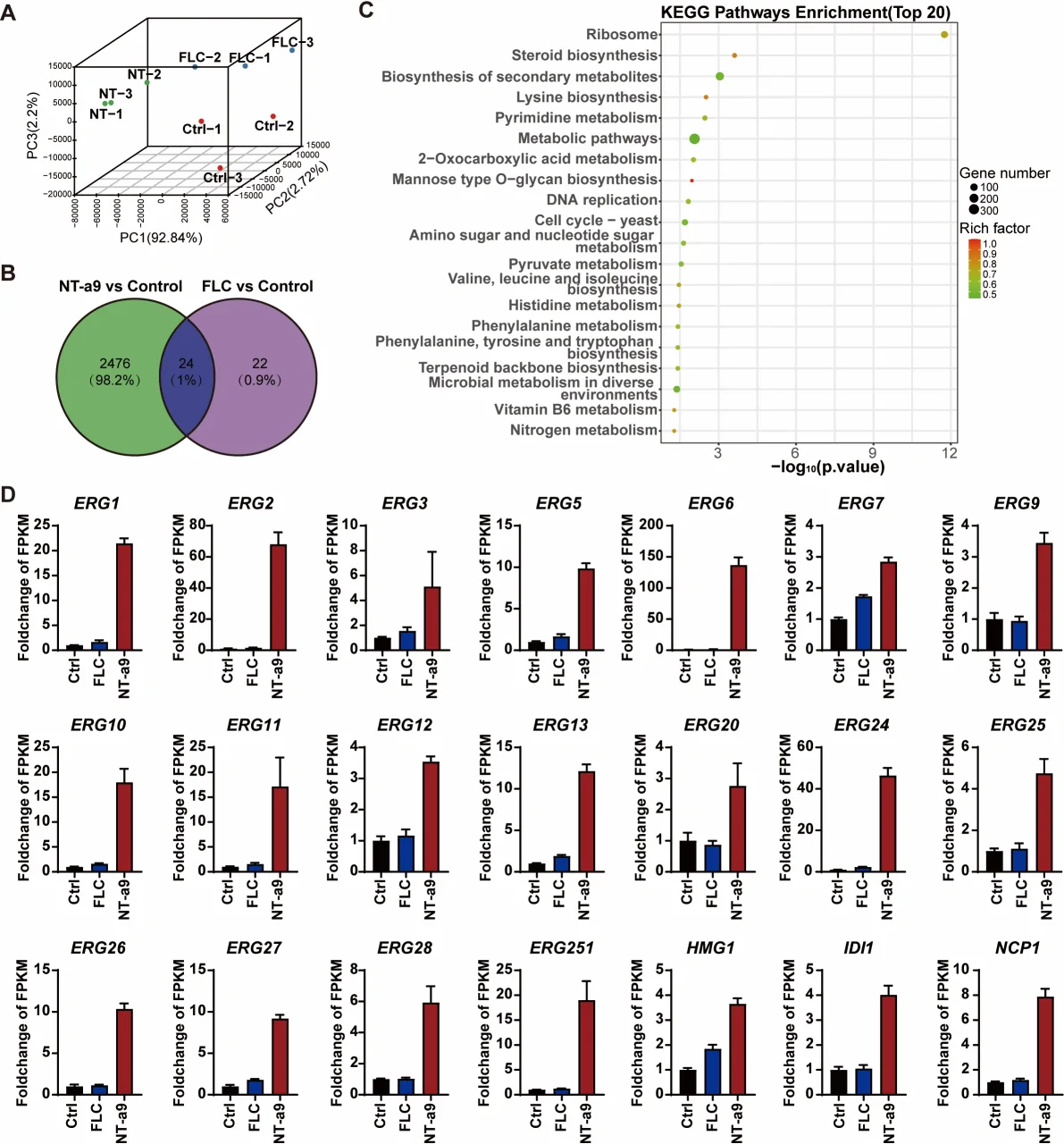
NT-a9 mainly inhibits sterol biosynthesis in *C. albicans***.** (**A**) The PCA of RNA-seq samples from *C. albicans* treated with or without drugs. (**B**) Venn diagrams of NT-a9 vs control and FLC vs control. (**C**) KEGG pathway enrichment of DEGs of the NT-a9 group compared to the control group. (**D**) The transcriptional level of genes involved in ergosterol biosynthesis, including *ERG1, ERG2, ERG3, ERG5, ERG6, ERG7, ERG9, ERG10, ERG11, ERG12, ERG13, ERG20, ERG24, ERG25, ERG26, ERG27, ERG28, ERG251, HMG1, IDI1*, and *NCP1*.

To further define the mechanistic differences between NT-a9 and FLC, we performed RNA sequencing (RNA-seq) in *C. albicans* (2 × 10⁶ CFU/mL) following 12 h of treatment with NT-a9 or FLC. Principal-coordinate analysis (PCoA) showed clear separation among treatment groups (Fig. 6A). At 4× MIC, FLC induced only 46 differentially expressed genes (DEGs), whereas NT-a9 altered expression of more than 2500 genes, indicating far more extensive transcriptional reprogramming (Fig. 6B). KEGG pathway enrichment confirmed that NT-a9 preferentially disrupted pathways related to sterol biosynthesis, consistent with our earlier findings (Fig. 6C). Notably, key ergosterol biosynthetic genes, including *ERG11, ERG6, ERG1*, and *ERG24*, were significantly upregulated in the NT-a9 group relative to both control and FLC-treated groups (Fig. 6D). This compensatory transcriptional response—especially upregulation of *ERG6*—drives the accumulation of toxic sterol intermediates (29), which further enhances the antifungal activity of NT-a9. Together, these results confirm that NT-a9 acts primarily by broadly disrupting ergosterol biosynthesis.

To evaluate the contribution of individual ergosterol biosynthetic genes to NT-a9 activity, we determined the MIC values of NT-a9 and FLC against a panel of *C. albicans* heterozygous (one-allele) and homozygous (two-allele) deletion mutants, as well as overexpression mutants, using SN152 as the parental reference (Fig. S2A and S2B). Relative to SN152, FLC MICs showed only minor fluctuations across most single-deletion strains. The *ERG9/erg9*Δ mutant exhibited increased susceptibility to both FLC and NT-a9. In striking contrast, NT-a9 displayed a dramatic reduction in MIC (≤ 0.001  µg/mL) against the *ERG11/erg11*Δ mutant, indicating that reduced Erg11 expression renders cells hypersensitive to NT-a9. Unexpectedly, NT-a9 MICs were increased against most other single-deletion strains, including *ERG2/erg2Δ, ERG3/erg3Δ, ERG6/erg6*Δ, and *ERG24/erg24*Δ, whereas FLC MICs remained unchanged. These observations support our model that compensatory upregulation of *ERG* genes under NT-a9 treatment promotes toxic sterol accumulation to augment antifungal activity; disruption of these genes (other than *ERG11*) relieves this synergistic effect and increases NT-a9 MICs.

A similar pattern was observed in double-deletion strains: FLC MICs remained stable, whereas NT-a9 MICs were consistently elevated, with the exception of the *erg3*Δ/Δ mutant, which cannot produce toxic sterols and showed reduced susceptibility to both agents. Among overexpression strains, *ERG11* overexpression exerted the most striking effect: it increased the NT-a9 MIC by 125-fold (from 0.004 μg/mL to 0.5 μg/mL) but raised the FLC MIC by only 8-fold (from 0.063 μg/mL to 0.5 μg/mL) relative to SN152 (Fig. S2A and S2B). This divergent shift strongly implies that much higher levels of Erg11 are required to offset NT-a9 binding compared with FLC. We therefore hypothesized that NT-a9 binds Erg11 with substantially higher affinity than FLC.

**Fig 7:**
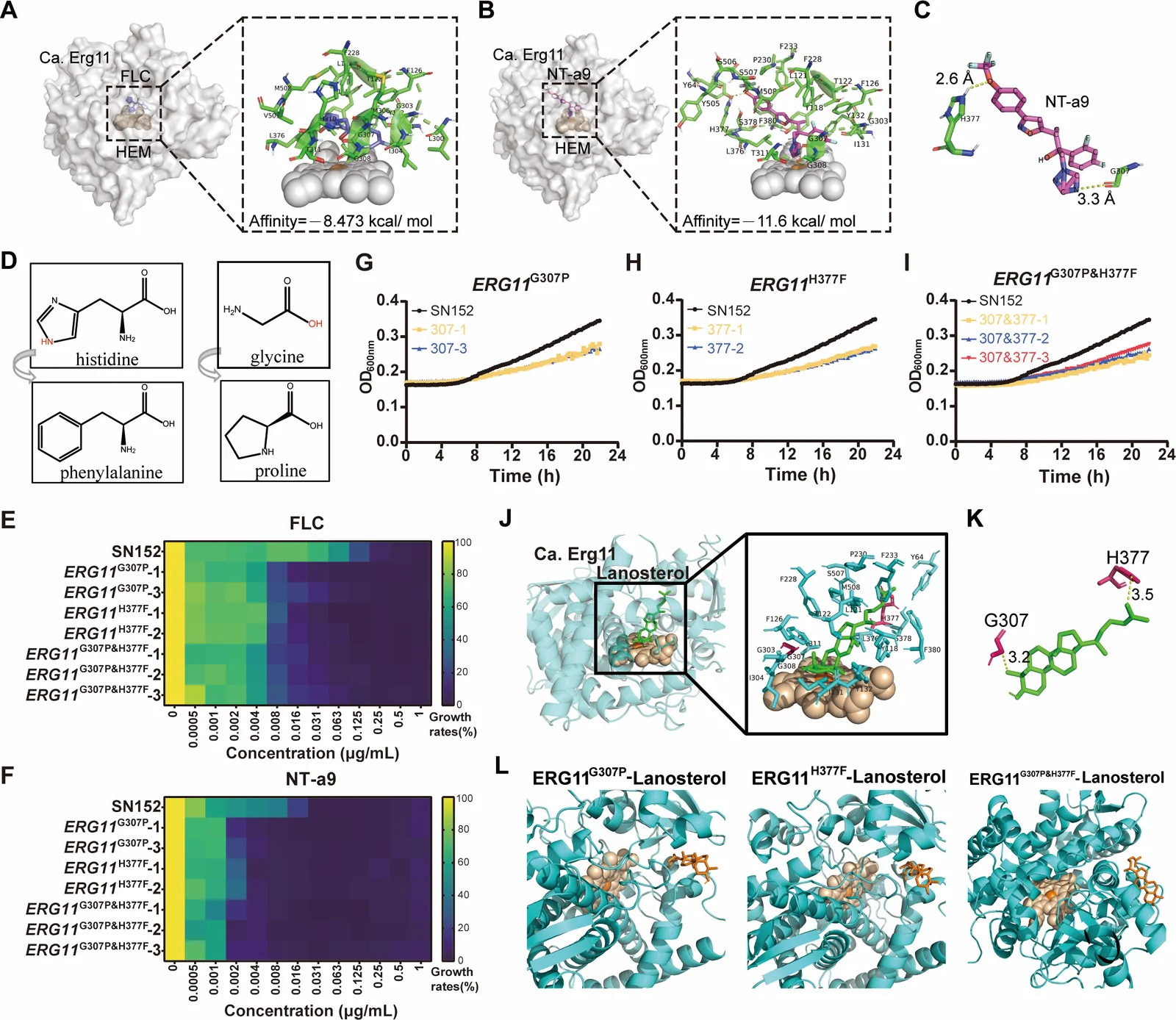
NT-a9 demonstrates high binding affinity to *C. albicans* Erg11. (**A and B**) Docking results of Ca. Erg11 with FLC or NT-a9. The purple structure represents FLC. The magenta structure represents NT-a9. The green stick structures indicate amino acid residues within 5 Å of the compound. The globular structure centered on the orange iron atom represents the heme-binding domain of Ca. Erg11, which is the catalytic site and the target of drug action. (**C**) The two amino acid sites interacting with NT-a9 are the carboxyl group of glycine at position 307 (G307) and the pyrrole-like nitrogen atom in the imidazole ring of histidine at position 377 (H377). (**D**) Schematic illustration of the site-directed substitutions: H377F (histidine to phenylalanine) and G307P (glycine to proline). (**E and F**) The MIC of FLC or NT-a9 against several amino acid point mutants using SN152 as the parental strain. Fungal growth rates are represented as heatmaps. (**G–I**) Time-growth curves of several amino acid point mutants. (**J**) Docking results of lanosterol with Ca. Erg11 protein. (**K**) Binding of lanosterol with specific G307 and H377 in Ca. Erg11. (**L**) Molecular docking of lanosterol with mutant Erg11 protein, with structure predicted by homology modeling with AlphaFold 3.

To test this hypothesis, we performed molecular docking using the *C. albicans* Erg11 crystal structure (PDB ID: 5FSA), with the heme group as the primary binding pocket. Docking simulations were conducted using AutoDock Vina (Fig. 7A through C). While both FLC and NT-a9 interact with Erg11, NT-a9 achieved a more favorable binding energy of −11.6 kcal/mol relative to FLC (−8.473 kcal/mol) (Fig. 7A and B). For human CYP51 (PDB ID: 6UEZ), NT-a9 yielded a binding energy of −6.028 kcal/mol (Fig. S3A). Consistent with this computational prediction, CCK-8 cytotoxicity assays demonstrated that NT-a9 exerts negligible toxic effects on human HUVEC and 293T cell lines (Fig. S3B). Within a 5 Å radius, 16 amino acid residues mediated FLC binding mainly through van der Waals forces, whereas 22 residues contributed to NT-a9 binding. Critically, NT-a9 formed direct hydrogen bonds with Gly307 and His377, whereas FLC interacted with Gly307 only weakly via van der Waals forces and did not engage His377 (Fig. 7C). These results identify Gly307 and His377 as key residues underlying the superior potency of NT-a9.

To further validate the critical roles of G307 and H377 in the binding of NT-a9 to Erg11, we performed single and double amino acid site-mutations at these sites. Given that H377 forms a hydrogen bond with NT-a9 through its imidazole ring (Fig. 7C), we substituted histidine with phenylalanine (Fig. 7D). G307 interacts with NT-a9 through its carboxyl group (Fig. 7C), a moiety not characteristic of glycine. Therefore, we substituted glycine, which has a flexible structure, with rigid proline to investigate the role of G307 in binding (Fig. 7D). To validate the functional importance of Gly307 and His377 in NT-a9–Erg11 binding, we constructed single and double amino acid substitution mutants: *erg11*^G307P^, *erg11*^H377F^, and *erg11*^G307P/H377F^, using SN152 as the parental strain (Fig. S3). All mutant strains exhibited increased susceptibility to both NT-a9 and FLC, with lower MIC values relative to SN152 (Fig. 7E and F). For the parental strain, the MIC ratio of FLC to NT-a9 was 16-fold (0.25 μg/mL vs 0.016 μg/mL). In all mutants, this ratio narrowed to only 4-fold (0.008 μg/mL vs 0.002 μg/mL). Although these mutations did not confer resistance, they selectively reduced the potency advantage of NT-a9 over FLC, confirming that Gly307 and His377 are more critical for NT-a9 binding than for FLC binding.

We next explored why these mutations increased drug susceptibility. Time-growth curves revealed that all mutant strains grew significantly slower than SN152 (Fig. 7G through I), indicating that Gly307 and His377 are essential for normal Erg11 function and fungal fitness. We hypothesized that these mutations impair lanosterol binding and thus reduce ergosterol synthesis. Molecular docking of lanosterol to wild-type Erg11 confirmed that Gly307 and His377 are important for substrate interaction, with distances of 3.2 Å and 3.5 Å, respectively (Fig. 7J and K). Homology modeling (AlphaFold 3) and docking with mutant Erg11 further demonstrated that substitutions at G307, H377, or both block lanosterol entry into the active site pocket and prevent access to the heme group (Fig. 7L), thereby inhibiting lanosterol demethylation and ergosterol production. Because both NT-a9 and FLC act by competing with lanosterol for Erg11 binding, these mutations compromise fungal growth and indirectly increase susceptibility to both agents, consistent with our MIC data.

In summary, Gly307 and His377 are essential for the catalytic function of Erg11 and for high-affinity binding of NT-a9. Mutations at these sites impair ergosterol biosynthesis, reduce fungal growth, and paradoxically enhance drug susceptibility, while specifically diminishing the superior potency of NT-a9 relative to FLC. These findings reveal a dual role of Gly307 and His377 in maintaining Erg11 enzymatic activity and mediating high-affinity interactions with NT-a9.

## DISCUSSION

In this study, we comprehensively characterized the novel triazole antifungal NT-a9 and demonstrated its potent, sustained, and broad-spectrum activity against pathogenic *Candida*, *Cryptococcus*, and *Aspergillus* species. *In vitro*, NT-a9 displayed strong antifungal activity against a large panel of clinical isolates, with potency significantly superior to FLC and comparable to VRC. More importantly, NT-a9 exerted durable growth inhibition and a prolonged PAFE against most *Candida* species, a feature rarely observed in conventional azoles. Notably, NT-a9 also exhibited a low risk of resistance development in tested fungal strains, overcoming a major shortcoming of currently available azoles. Mechanistically, NT-a9 markedly suppresses ergosterol biosynthesis, triggers severe fungal membrane damage, and ultimately induces cell death. In murine models of invasive candidiasis, low-dose NT-a9 conferred remarkable survival benefits and effectively reduced renal fungal burden.

A major limitation of currently available azoles is their low genetic barrier to resistance, which allows rapid emergence of resistant mutants during therapy (17, 30, 31). In contrast, NT-a9 displayed an exceptionally high resistance barrier. It showed that low MPC values and fully suppressed resistance development in *Cryptococcus* species. Moreover, in 28-day serial passage assays, *C. albicans* did not develop detectable resistance to NT-a9, in sharp contrast to the significant resistance induced by FLC. NT-a9 also retained potent activity against FLC-resistant *C. neoformans* strains, including those overexpressing drug efflux pumps, indicating minimal cross-resistance and limited impact from major azole resistance mechanisms. Of note, few approved azoles exert reliable activity against multidrug-resistant *C. auris,* an emerging pathogen that poses a severe global health threat. Our *in vivo* experiments confirmed that NT-a9 achieves robust therapeutic efficacy against this difficult-to-treat fungus. Collectively, these properties enable NT-a9 to address critical unmet clinical needs for treating drug-resistant IFIs and support its potential for long-term clinical applications.

Another key unmet need addressed by NT-a9 is the lack of durable activity and prolonged PAFE among approved azoles. Most clinically used azoles exert only transient fungal suppression, requiring frequent dosing and limiting sustained efficacy (14, 32, 33). In contrast, NT-a9 produced long-lasting growth inhibition and a significantly prolonged PAFE in major *Candida* pathogens. This sustained pharmacodynamic action reduces the risk of fungal regrowth after drug elimination and supports less frequent dosing regimens, representing a clear advantage over conventional azoles.

Mechanistically, we demonstrated that NT-a9 acts by strongly inhibiting ergosterol biosynthesis through high-affinity binding to the target enzyme Erg11 (34). Similar to classical azoles, NT-a9 interferes with lanosterol 14α-demethylation by interacting with the heme iron of Erg11. However, molecular docking revealed that unlike FLC, NT-a9 forms important hydrogen bonds with Gly307 and His377 within the Erg11 active site, which contributes to its improved binding affinity in this structural model. These specific interactions enable more potent inhibition of ergosterol production, increased toxic sterol accumulation, severe membrane damage, and ultimately enhanced fungal killing. Although His377 has been implicated in drug binding in other fungal pathogens (35), this study is the first to clarify the physiological functions of both Gly307 and His377 in *C. albicans* and to identify Gly307 as a novel key site mediating antifungal agent binding to Erg11. These insights provide a novel structural basis for the design and optimization of high-potency azoles.

Several limitations of this study should be noted. First, all assays for antifungal activity and resistance development were conducted *in vitro* or in murine infection models. The results from simplified laboratory systems may not reflect the complex host microenvironment in human patients, restricting the clinical translatability of our observations. Second, the interaction between NT-a9 and Erg11 was primarily predicted via *in silico* molecular docking. In the absence of direct structural evidence from protein-drug co-crystallization, the simulated binding mode, hydrogen bond networks, and key residue interactions require further experimental validation. Third, our investigation into Gly307 and His377 was confined to phenotypic analyses such as susceptibility tests and growth profiling. Biochemical experiments measuring enzyme activity and binding affinity are necessary to define their precise biological functions.

In conclusion, NT-a9 shows potent and sustained activity, a high genetic barrier to resistance, prolonged PAFE, and excellent efficacy against FLC-resistant pathogens including *C. auris*. Mechanistically, its high-affinity binding to Erg11 via Gly307 and His377 underlies its superior antifungal potency. NT-a9 represents a novel triazole that overcomes multiple key limitations of currently available azoles and is a promising new agent for the treatment of IFIs.

## METHODS AND MATERIALS

### Agents, primers, strains, and cultural conditions

All fungal strains used for antifungal susceptibility evaluation were predominantly clinical isolates. Reference strains including *C. albicans* SC5314, *C. neoformans* H99, *C. parapsilosis* ATCC 22019, and *C. krusei* ATCC 6258 were purchased from the Institute of Dermatology, Chinese Academy of Medical Sciences. Isogenic strains including *C. albicans* SN152, *ERG11*/*erg11Δ*, P-*ERG11*, and FLC-resistant *C. neoformans* strains (AFR1-OE, TCZ-1, TCZ-2, BFA-1, and BFA-2), as well as a full panel of *C. albicans* ergosterol biosynthesis mutant strains, were sourced from our in-house strain collection. Site-specific mutant strains generated in this study, including *erg11*^G307P^, *erg11*^H377F^, and *erg11*^G307P/H377F^, were constructed and validated using primer pairs listed in Tables S1 and S2. All fungal stocks were preserved in 40% glycerol and stored at −80°C for long-term maintenance.

RPMI 1640 broth medium was formulated with 10.0 g/L RPMI 1640 powder (Gibco, 31800022) and 34.5 g/L 3-(N-morpholino)propanesulfonic acid (MOPS; Diamond, A100670-0500), and the pH was adjusted to 7.0. The prepared medium was sterilized via filtration through 0.22-μm membrane filters (Beyotime, FVF025). For routine culture and activation, all yeast strains were passaged twice on Sabouraud dextrose agar (SDA) plates containing 1% peptone, 4% dextrose, and 1.8% agar at 30°C for 48 h. Single colonies were then inoculated into yeast extract-peptone-dextrose (YPD) liquid medium and incubated with shaking overnight at 30°C for 16 h to obtain logarithmic-phase fungal cultures for subsequent experiments.

NT-a9, VRC, and FLC were provided by Yangtze River Pharmaceutical Group. Stock solutions were serially prepared at concentrations of 102.4, 25.6, 6.4, 1.6, and 0.4 mg/mL using dimethyl sulfoxide (DMSO; BBI, A600163-0250). To eliminate solvent interference, the final DMSO concentration in all experimental systems was maintained at ≤1% (vol/vol).

### Construction of *ERG11* mutants

*Erg11* point mutants of *C. albicans*, including two single-site mutants (*erg11*^G307P^ or *erg11*^H377F^) and one double-site mutant (*erg11*^G307P/H377F^), were constructed via homologous recombination according to previously reported methods with minor modifications (36). Briefly, three recombinant plasmids harboring individual mutated *ERG11* sequences were chemically synthesized. The helper plasmids pSN52 (carrying the *HIS1* selection marker) and pSN69 (carrying the *ARG4* selection marker) were extracted using a standard plasmid purification kit. Target fragments, including mutated *ERG11* coding sequences, *ERG11* downstream homologous arm sequences, and selectable marker genes (*HIS1* or *ARG4*), were amplified by PCR using primer pairs containing homologous recombination overlap sequences. All PCR products were purified with a DNA purification kit. Subsequently, the mutated *ERG11* fragments were separately fused with *HIS1* and *ARG4* marker genes via fusion PCR.

The PEG/lithium acetate yeast transformation method combined with a commercial yeast transformation kit was applied for strain engineering. First, the *ERG11-HIS1* fusion cassette was transformed into the parental *C. albicans* SN152 strain to replace one native *ERG11* allele through homologous recombination. Positive transformants were screened on histidine-deficient selective plates and further verified by PCR amplification. The validated heterozygous mutant strain was subsequently used for the second round of transformation, in which the *ERG11-ARG4* fusion cassette was introduced to replace the remaining native *ERG11* allele. After screening on selective plates lacking both histidine and arginine, candidate strains were confirmed by PCR genotyping. Strains with correct point mutations in the *ERG11*-coding region were regarded as successfully constructed homozygous mutant strains.

### MIC determination

MICs were determined via the broth microdilution method in accordance with the Clinical and Laboratory Standards Institute (CLSI) M27 Ed4 guidelines for yeasts and CLSI M38 Ed3 guidelines for filamentous fungi, with minor modifications. Briefly, *Candida* and *Cryptococcus* strains were cultured on SDA plates at 30°C for 48 h, while *Aspergillus* strains were incubated on SDA plates at 28°C for 72 h.

For yeast inoculum preparation, single colonies of *Candida* and *Cryptococcus* species were resuspended in RPMI 1640 medium, and the cell turbidity was adjusted to 0.6 McFarland standard using a densitometer (bioMérieux, DensiCHEK, Germany). The fungal suspension was further diluted with RPMI 1640 medium to obtain a working inoculum of 0.5–2.5 × 10^3^ cells/mL. For *Aspergillus* species, spores were harvested and suspended in RPMI 1640 medium supplemented with glass beads, vigorously vortexed, and allowed to stand for 3 min. The supernatant was collected, adjusted to 0.5 McFarland standard, and diluted to a working inoculum of 0.2–2.5 × 10^4^ cells/mL.

Subsequently, equal volumes of fungal working inoculum and 2-fold serially diluted drug solutions were added to 96-well microtiter plates. Drug-free wells containing only fungal inoculum were set as positive growth controls. Plates were incubated under strain-specific conditions: 24 h at 30°C for *Candida* species, 48 h at 30°C for *Cryptococcus* species, and 72 h at 28°C for *Aspergillus* species. The MIC was defined as the lowest drug concentration that substantially reduced fungal growth turbidity relative to the positive control. The MIC was defined as the minimum drug concentration required to inhibit the growth of 50% of tested fungal isolates of a given species.

MIC assays for specific strains, including *C. albicans* SN152, *ERG11*/*erg11*Δ, P-*ERG11*, FLC-resistant *C. neoformans* (AFR1-OE, TCZ-1, TCZ-2, BFA-1, and BFA-2), and *C. albicans* site-specific mutants (*ERG11*, *ERG11*, and *ERG11*), were performed following the identical protocol described above. Fungal growth abundance was quantified by measuring the optical density at 600 nm (OD_600_) using a microplate reader (Thermo Fisher Scientific, Multiskan Sky, USA). Growth profiles of different strains were finally visualized as a heatmap generated with GraphPad Prism 9 software.

### Time-growth curve assay

To dynamically evaluate the antifungal activity of NT-a9 and clinical azoles against different fungal strains, time-growth curve assays were conducted according to previously described methods with minor modifications (37). Tested compounds were serially diluted in YPD medium to achieve 4-fold concentrated working solutions, and 50 μL of each drug solution was transferred into 96-well microtiter plates. Drug-free YPD medium was included as a negative control, with three biological replicates set for each group. Overnight cultured fungal suspensions were diluted to a final density of 1 × 10^4^ cells/mL, and 150 μL of the standardized inoculum was added to each well. Plates were incubated at 30°C for 48 h in a microplate reader (TECAN, Infinite F200 PRO, Switzerland), and the OD_600_ was automatically measured at 2-h intervals. Fungal growth curves were generated using GraphPad Prism 9 software, with incubation time on the x-axis and OD_600_ values reflecting fungal growth abundance on the y-axis.

### PAFE determination

PAFE assays were performed according to previously established protocols with minor modifications to evaluate the residual antifungal activity of NT-a9 and FLC after drug removal (32, 38). Briefly, overnight-cultured fungal cells were washed three times with sterile phosphate-buffered saline (PBS; Sangon Biotech, B040100-0005) and resuspended in RPMI 1640 medium to prepare a working inoculum with an OD_600_ of 0.3. For drug treatment, 50 μL of NT-a9 or FLC stock solution was added to 5 mL of fungal inoculum to yield a final drug concentration of 16 μg/mL, while the control group was treated with an equal volume of DMSO. All samples were incubated at 30°C for 4 h with continuous shaking at 200 rpm. Following incubation, residual drugs were completely removed via three rounds of centrifugation (8,000 rpm, 3 min) and PBS washing. The harvested cell pellets were resuspended in fresh RPMI 1640 medium, and the cell density was standardized to a consistent OD_600_ ranging from 0.10 to 0.15. A 200 μL aliquot of each standardized fungal suspension was transferred into individual wells of a 96-well microtiter plate, with three biological replicates prepared for each experimental group. Plates were incubated at 30°C for 24 h in a TECAN microplate reader, and the OD_600_ values were automatically recorded at 15-min intervals. The PAFE value was calculated using the formula: PAFE = T − C. In this equation, T represents the time required for drug-treated fungal cells to double their initial OD_600_ value, and C indicates the corresponding doubling time of the untreated control group. Both T and C values were determined by nonlinear logistic curve fitting of OD_600_–time data using Origin software.

### MPC determination

MPC assays were carried out according to previously reported methods with minor modifications (39). SDA plates containing serially diluted concentrations of NT-a9 or FLC were prepared in advance for resistance screening. Fungal strains were twice activated on SDA plates, and a single fresh colony was inoculated into 10 mL of YPD medium, followed by overnight incubation at 30°C with constant shaking at 200 rpm for 16 h. The logarithmic-phase fungal suspension was centrifuged at 5,000 rpm for 1 min, washed twice with sterile PBS, and adjusted to a cell density ranging from 2 × 10^8^ to 2 × 10^7^ cells/mL. A 100 μL aliquot of the standardized fungal suspension was evenly spread onto each pre-prepared drug-containing SDA plate. Plates were incubated at 30°C for up to 5 days, with daily growth observation. The MPC was defined as the lowest drug concentration that completely prevented visible fungal colony formation on SDA plates throughout the 5-day incubation period.

### *In vitro* antifungal resistance evolution via serial passaging

*In vitro* experimental evolution assays were performed according to previously described protocols with minor modifications to evaluate the dynamic resistance development of *C. albicans* SC5314 under continuous NT-a9 and FLC pressure (40). All experimental data for NT-a9 were acquired concurrently with parallel FLC control groups, consistent with our previously reported study design. Briefly, overnight-cultured fungal suspensions were standardized to 2 × 10^6^ cells/mL in fresh RPMI 1640 medium and incubated with NT-a9 or FLC at a concentration of 2-fold the baseline MIC, while drug-free medium served as the negative control. All samples were incubated at 37°C for 24 h with continuous shaking at 200 rpm, and fungal growth was quantified by measuring the OD_600_ value after incubation. Subsequent passage conditions were determined based on the relative growth of drug-treated groups compared with the untreated control group. If the OD_600_ of the drug-exposed group exceeded 50% of the drug-free control value, the fungal suspension was transferred into fresh medium with a 2-fold increased drug concentration. In contrast, if the OD_600_ of the drug-treated group was lower than 50% of the control value, fungal cells were passaged into fresh medium containing the original drug concentration. Every 24 h, fungal cultures were diluted and spread onto SDA plates for 48 h of incubation. Three single colonies were randomly selected from each group for MIC detection. The serial passage experiment lasted for 28 consecutive days, and the dynamic changes in MIC values for each treatment group were recorded and visualized using GraphPad Prism 9 software.

### Mouse model of invasive candidiasis caused by *C. albicans* and *C. auris*

All animal experiments were conducted in accordance with previously established protocols with minor modifications (41, 42). Female ICR mice aged 7–8 weeks were purchased from Shanghai SLAC Laboratory Animal Corporation (Shanghai, China). All animal experimental procedures were approved by the Animal Care Committee of Tongji University (approval No. TJAA00325102), and all institutional and national guidelines for the care and humane use of laboratory animals were strictly followed.

For the establishment of the *C. albicans* invasive candidiasis model, overnight-cultured *C. albicans* SC5314 cells were washed three times with sterile normal saline and standardized to a final concentration of 1 × 10^7^ cells/mL. Each mouse was inoculated with 200 μL of the fungal suspension via tail vein injection. At 24 h post-infection, mice received a single oral gavage of NT-a9 (0.25 mg/kg or 1 mg/kg) or FLC (0.5 mg/kg or 2 mg/kg). Mice in the vehicle control group were administered an equal volume of 0.5% sodium carboxymethylcellulose (CMC-Na; Sangon Biotech, A501427-0250), which served as the drug solvent. Each experimental group contained 10 mice, and animal survival was monitored and recorded daily for 28 consecutive days. Survival curves were generated using GraphPad Prism 9 software, and statistical differences among groups were analyzed via the Log-rank (Mantel-Cox) test.

To assess *in vivo* fungal burden, mice were anesthetized and euthanized on day 3 post-infection, and kidney tissues were aseptically harvested. The right kidney was weighed and homogenized in sterile normal saline. The tissue homogenate was serially diluted in 10-fold gradients, spread onto SDA plates, and incubated at 30°C for 48 h for colony counting. Fungal loads were calculated and expressed as colony-forming units (CFU) per gram of kidney tissue, with six biological replicates per group. Quantitative data were visualized in GraphPad Prism 9, and statistical significance was determined using one-way analysis of variance (ANOVA).

The left kidney tissues were fixed in 4% paraformaldehyde (PFA; Sangon Biotech, E672002-0100) at 4°C for 24 h. After thorough PBS rinsing, tissues were dehydrated, paraffin-embedded, and sectioned into 3 μm-thick slices using a microtome. Tissue sections were stained with periodic acid-Schiff (PAS) stain and observed under an inverted fluorescence microscope (NIKON, ECLIPSE CI-S, Japan). Histological images were processed and exported using KF-Viewer software (v1.7.0.24) for subsequent pathological analysis.

For the *C. auris* invasive infection model, mice were immunocompromised by intraperitoneal injection of cyclophosphamide (200 mg/kg; Yuanye Biotechnology, S30563-25g) 3 days prior to fungal inoculation, with a supplementary dose administered 1 day post-infection to maintain immunosuppression. Overnight-cultured *C. auris* isolate 278 cells (CBS 12768, Clade I) were harvested, washed three times with sterile normal saline, and adjusted to a concentration of 1 × 10^8^ cells/mL. Each immunosuppressed mouse (*n* = 10 per group) received a 200 μL tail vein injection of the standardized fungal suspension. At 4 h post-infection, mice were treated with a single oral gavage of NT-a9 (10 mg/kg or 20 mg/kg), FLC (10 mg/kg or 20 mg/kg), or 0.5% CMC-Na vehicle control. All animals were monitored daily for 21 days. Survival curves were plotted using GraphPad Prism 9, and intergroup statistical differences were evaluated using the Log-rank (Mantel-Cox) test.

### Checkerboard combination assay

Checkerboard combination assays were carried out according to previously reported methods with minor modifications to explore the combinatorial antifungal effects of NT-a9 or FLC with exogenous ergosterol (BBI, A600443-0025) against *C. albicans* SC5314 (43, 44). All experiments were performed in 96-well microtiter plates with strictly configured gradient systems and control groups. Briefly, column 1 was set as the drug-free control gradient and row A as the ergosterol-free control gradient, with 50 μL of RPMI 1640 medium added to each corresponding well. Wells A12–D12 served as blank negative controls and were filled with 200 μL of RPMI 1640 medium per well, whereas wells E12–H12 and well A1 were designated as fungal growth positive controls, each containing 100 μL of RPMI 1640 medium. For gradient preparation, 2-fold serially diluted NT-a9 or FLC solutions were dispensed into columns 2–8 at 50 μL per well, with concentrations increasing progressively from left to right. Similarly, 2-fold serially diluted ergosterol solutions were added to rows B to H at 50 μL per well, with concentrations increasing gradually from top to bottom. Overnight-cultured *C. albicans* SC5314 suspensions were standardized to a working inoculum of 2–3 × 10^3^ cells/mL, and 100 μL of the calibrated fungal suspension was added to all experimental wells, excluding the blank control wells (A12–D12). The assembled plates were incubated at 30°C for 24 h. Fungal growth abundance was quantified by detecting the optical density at 600 nm (OD). Finally, the combinatorial growth profiles were processed and visualized as a heatmap using GraphPad Prism 9 software.

### Ergosterol biosynthesis quantification via GC-MS

The regulatory effects of NT-a9 on ergosterol biosynthesis in *C. albicans* were determined according to previously described protocols with minor modifications (43, 45). Briefly, 1 mL of overnight-cultured fungal suspension was inoculated into 100 mL of YPD medium, followed by treatment with gradient concentrations of NT-a9 or FLC; drug-free YPD cultures were set as the untreated control. After 12 h of shaking incubation at 30°C and 200 rpm, fungal cells were harvested and rinsed three times with sterile deionized water, and the wet weight of each cell pellet was standardized to 0.7 g for consistent quantification. For sterol saponification and extraction, each standardized cell sample was supplemented with 2.5 mL of sterile water and 6 mL of freshly prepared 15% sodium hydroxide solution (Sangon Biotech, A100173-0500) dissolved in 90% ethanol (Sangon Biotech, A500737-0500). After thorough mixing, 180 μg of cholesterol (Diamond, A100433-0025) dissolved in n-hexane (Sangon Biotech, A530007-0500) was added to each sample as an internal standard. The mixtures were incubated in an 80°C water bath for 1 h to complete saponification. Total sterols were subsequently extracted three times with 6 mL of petroleum ether (Sinopharm, 10015218); each extraction involved vigorous vortexing for 2 min followed by a 3 min static settlement. All petroleum ether layers from repeated extractions were collected and pooled and then washed twice with sterile deionized water. The organic solvent was evaporated in a 65°C water bath for 20 min. The residual sterol extracts were finally redissolved in 600 μL of n-hexane, and a 200 μL aliquot was subjected to GC–MS detection (Thermo Fisher Scientific, Trace DSQ II, USA) using an HP-50 gas chromatography column (Agilent, USA).

Raw chromatographic peak areas were acquired from GC data, and sterol structural identification was verified based on corresponding mass spectrometry fragment information. The relative content of individual sterols was calculated as the peak area ratio of each target sterol to the internal standard cholesterol and further normalized to the wet weight of fungal cell pellets. The proportional percentage of each sterol component in the total sterol pool was subsequently quantified. Of note, the absolute retention time of identical sterol compounds may vary across different samples and experimental conditions. Therefore, definitive identification of sterol components was strictly determined by characteristic MS fragment ion recognition rather than retention time alone.

### Flow cytometric analysis of cell membrane integrity

Flow cytometric assays were performed according to previously established methods with minor modifications to evaluate fungal cell membrane damage induced by NT-a9 and FLC (46). Briefly, *C. albicans* SC5314 suspensions at a density of 2 × 10^6^ cells/mL were treated with NT-a9 or FLC at concentrations of 1× MIC, 4× MIC, and 16× MIC. Drug-free cultures were set as the blank control group. All samples were incubated at 30°C with continuous shaking at 200 rpm for 3.5 h or 6 h. After incubation, fungal cells were harvested and stained with propidium iodide (PI; 10 μg/mL in PBS; Sigma-Aldrich, P4170) at 30°C for 30 min in the dark. Following PI staining, the cells were rinsed with sterile PBS and fixed with 4% PFA for subsequent detection.

Flow cytometry detection was performed with standardized parameter settings. PI fluorescence signals were acquired via the phycoerythrin (PE) channel, with an excitation wavelength of 561 nm and an emission wavelength of 585 ± 15 nm. The forward scatter (FSC) and side scatter (SSC) voltages were set to 250 V and 280 V, respectively, to accurately gate intact *C. albicans* cells and eliminate interfering cell debris. A total of 10,000 cellular events were collected for each sample to meet the data analysis criteria. All raw flow cytometry data were analyzed using FlowJo V10 software to generate cell fluorescence distribution histograms and calculate the mean fluorescence intensity (MFI) values.

### Transmission electron microscopy analysis

TEM analysis was performed according to previously reported methods with minor modifications to observe the ultrastructural morphological changes of *C. albicans* induced by NT-a9 and FLC treatment (43, 47). Briefly, *C. albicans* SC5314 suspensions at a density of 2 × 10^6^ cells/mL were incubated with NT-a9 or FLC at gradient concentrations of 1× MIC, 4× MIC, and 16× MIC for 6 h at 30°C with continuous shaking at 200 rpm. Drug-free fungal cultures were included as the untreated control. After incubation, fungal cells were harvested, rinsed with sterile PBS, and thoroughly fixed with electron microscopy fixative (Servicebio, G1102) at 4°C for 24 h. The fixed cell pellets were collected by centrifugation and washed three times with 0.1 M PBS (pH 7.4) and then embedded in 1% agarose solution. The obtained agarose blocks were post-fixed with 1% osmium tetroxide (OsO_4_; Ted Pella Inc., 18456) diluted in 0.1 M phosphate buffer (PB) for 2 h at room temperature in the dark. Following complete removal of residual OsO_4_ and thorough PB rinsing, samples were subjected to gradient dehydration using a series of ethanol solutions (Sinopharm, 100092183) with increasing concentrations (30%, 50%, 70%, 80%, 95%, 100%, and 100%), with 20 min incubation for each gradient. Samples were further dehydrated twice with 100% acetone (Sinopharm, 10000418), 15 min per treatment.

Subsequently, the dehydrated samples were processed via resin infiltration, embedding, and polymerization. The prepared resin blocks were ultrathin-sectioned into 60–80 nm slices using an ultramicrotome (Leica UC7, Germany). The ultrathin sections were double-stained with 2% saturated uranium acetate alcoholic solution (Ted Pella Inc., 18456) for 8 min in the dark, followed by 2.6% lead citrate solution (prepared from lead nitrate (Sigma, 203580) and trisodium citrate (Sinopharm, 10019408)) for 8 min under CO_2_-free conditions. After overnight drying at room temperature, the cellular ultrastructure was observed and imaged using a transmission electron microscope (HITACHI, HT7800, Japan).

### Transcriptome RNA-seq analysis

Transcriptomic profiling was performed according to previous procedures with minor modifications to investigate global gene expression changes in *C. albicans* SC5314 in response to NT-a9 and FLC treatment (48, 49). Briefly, overnight-cultured *C. albicans* SC5314 suspensions were standardized to 2 × 10^6^ cells/mL and incubated with NT-a9 (4× MIC), FLC (4× MIC), or drug-free medium at 30°C with continuous shaking at 200 rpm for 12 h. Each experimental group was prepared in three biological replicates. For each sample, 1 mL of fungal culture was collected, and total RNA was extracted using the Column Fungal RNA Extraction Kit (KANLANG, KL-10112BTN) under strict sterile and RNase-free conditions. The obtained RNA samples were immediately snap-frozen in liquid nitrogen for subsequent quality verification and library preparation.

RNA integrity and quality were evaluated using the Agilent 4150 system, and only high-quality samples that met the library construction criteria were retained for cDNA library generation. Whole-transcriptome sequencing was performed on the DNBSEQ-T7 platform (MGI Tech). Raw sequencing reads were subjected to strict quality control and filtered to remove low-quality reads and adapter sequences, yielding clean reads for subsequent bioinformatic analysis. The processed clean reads were mapped to the *C. albicans* SC5314 reference genome using HISAT2 software. Gene expression levels were quantified via FeatureCounts, and fragments per kilobase of transcript per million mapped reads (FPKM) values were calculated to normalize and compare transcriptional abundance across all samples.

Differentially expressed genes (DEGs) were identified using the DESeq2 algorithm, with the screening thresholds set as adjusted *P*-value (padj) < 0.05 and |log2(fold change)| >1. Functional enrichment analyses of DEGs, including Gene Ontology (GO) term enrichment and Kyoto Encyclopedia of Genes and Genomes (KEGG) pathway enrichment, were further performed to annotate the biological functions and signaling pathways associated with NT-a9 and FLC responsiveness in *C. albicans*.

### Molecular docking analysis

Molecular docking simulations were performed according to standard computational protocols with minor modifications using AutoDockTools 1.5.7 and AutoDock Vina software to characterize the binding interactions of candidate compounds with *C. albicans* Erg11 or homo CYP51 (50). In all docking simulations, small-molecule ligands were defined as flexible structures, while the target protein was set as a rigid structure. The crystal structures of *C. albicans* Erg11 (PDB ID: 5FSA) and Homo CYP51 (PDB ID: 6UEZ) were retrieved from the Research Collaboratory for Structural Bioinformatics Protein Data Bank (RCSB PDB). For proteins without available crystal structures, three-dimensional structural models were predicted using AlphaFold 3 (alphafoldserver.com).

Prior to molecular docking, all redundant polymer chains, exogenous ligands, and water molecules were removed from the protein crystal structure using PyMOL software. The three-dimensional molecular structures of NT-a9, FLC, and lanosterol were constructed via ChemDraw 20.0, and molecular energy minimization was subsequently performed using Chem3D 20.0 to optimize the spatial conformation of each ligand. For structure pretreatment, polar hydrogen atoms were added to both the protein and ligand molecules, and Gasteiger partial charges were calculated. Flexible torsion angles of the ligands were defined through the torsion tree module, and all processed structural files were converted into PDBQT format for subsequent docking calculations.

The grid box was centrally positioned on the iron atom within the CYP51 active pocket, with dimensional parameters set to 60 × 50 × 54 Å. Binding affinity values were calculated using the default scoring function of AutoDock Vina. The optimal docking conformation with the lowest binding energy was selected as the final docking result. Molecular binding modes and interaction profiles between the target protein and small-molecule ligands were visualized and analyzed using PyMOL software.

### Cytotoxicity tests

Human umbilical vein endothelial cells (HUVECs) and Human Embryonic Kidney 293T cells (293T) were maintained in DMEM (HyClone, SH30243.01) supplemented with 10% FBS (Sigma-Aldrich, F0193). CCK-8 assays were applied to test compound cytotoxicity. Briefly, 1 × 10⁴ HUVECs or 293T cells were seeded per 96-well well and incubated at 37°C for 3 h to attach. The culture medium was replaced with serum-free DMEM containing serially diluted NT-a9 (1–64 μg/mL), followed by a 24-h incubation; 10 μL of CCK-8 reagent (BIOAGRIO, CK192-1500) was added to each well, and the plates were incubated for another 2 h at 37°C before measuring absorbance at 450 nm on an ELISA reader. DMSO-treated cells (final solvent < 1% vol/vol) were used as the 100% viability control.

## Data Availability

The raw RNA-seq data reported in this study are deposited in the NCBI Gene data sets under accession number GSE297321.

## SUPPLEMENTAL MATERIAL

Fig. S1 aac.00768-26-s0001.docx Mouse model of invasive fungal infections.

Fig. S2 aac.00768-26-s0002.docx MICs of FLC and NT‑a9 against *C. albicans* strains with gene deletions or overexpression in the ergosterol biosynthesis pathway.

Fig. S3 aac.00768-26-s0003.docx The toxicological evaluation of NT-a9.

Table S1 aac.00768-26-s0004.xlsx Strains were used in this study.

Table S2 aac.00768-26-s0005.xlsx Primers were used in this study.

ASM does not own the copyrights to Supplemental Material that may be linked to, or accessed through, an article. The authors have granted ASM a non-exclusive, world-wide license to publish the Supplemental Material files. Please contact the corresponding author directly for reuse.
