# Supplementary material for: NT-A9, a new triazole, exhibits potent antifungal activity against Candida and Cryptococcus species through potent ergosterol biosynthesis inhibition
Source: Antimicrob Agents Chemother. 2026 Aug 5;70(9):e00768-26. doi: 10.1128/aac.00768-26 (PMC13712951; doi:10.1128/aac.00768-26)
Supplement: Supplementary file 1 [file aac.00768-26-s0001.docx]

**Figure S1. Mouse model of invasive fungal infections.** (A) Mouse model of invasive *C. albicans* infection and experimental procedure. *C. albicans* SC5314 (2×10⁶ cells/mouse) was injected into ICR mice via the tail vein on Day 0. At 24 hours post-infection, the drug (NT-a9 or FLC) was administered by oral gavage (single treatment). On Day 3 post-infection, mouse kidneys were collected for renal fungal load quantification and histopathological analysis. Mouse survival was monitored daily for 28 days. (B) Mouse model of invasive *C. auris* infection and experimental procedure. Prior to infection, ICR mice were immunosuppressed through intraperitoneal injection of cyclophosphamide (200 mg/kg in PBS) administered at two time points: three days pre-infection and one day post-infection. On the day of infection, *C. auris* 278 (2×10^7^ cells/mouse) was injected into immunosuppressed mice via the tail vein on Day 0. At 4 hours post-infection, the drug (NT-a9 or FLC) was administered by oral gavage (single treatment). Mouse survival was monitored daily for 21 days.
