# Supplementary material for: NT-A9, a new triazole, exhibits potent antifungal activity against Candida and Cryptococcus species through potent ergosterol biosynthesis inhibition
Source: Antimicrob Agents Chemother. 2026 Aug 5;70(9):e00768-26. doi: 10.1128/aac.00768-26 (PMC13712951; doi:10.1128/aac.00768-26)
Supplement: Supplementary file 2 [file aac.00768-26-s0002.docx]

**Figure S2. MICs of FLC and NT‑a9 against *C. albicans* strains with gene deletions or overexpression in the ergosterol biosynthesis pathway**. The heatmap shows MIC values (μg/mL) for FLC (A) and NT‑a9 (B) against single‑knockout (green), double‑knockout (blue), and overexpression (red) strains, with SN152 as the parental control. Data are presented as the mode of at least three independent experiments. The red star indicates which strain exhibits the greatest change in MIC for NT‑a9 compared to SN152.
