# Supplementary material for: NT-A9, a new triazole, exhibits potent antifungal activity against Candida and Cryptococcus species through potent ergosterol biosynthesis inhibition
Source: Antimicrob Agents Chemother. 2026 Aug 5;70(9):e00768-26. doi: 10.1128/aac.00768-26 (PMC13712951; doi:10.1128/aac.00768-26)
Supplement: Supplementary file 3 [file aac.00768-26-s0003.docx]

**Figure S3.** The toxicological evaluation of NT-a9. (A) NT-a9 demonstrates weaker binding affinity to Homo CYP51. Docking results of Homo CYP51 with NT-a9. The magenta structure represents NT-a9. The green stick structures indicate amino acid residues within 5 Å of the compound. The globular structure centered on the orange iron atom represents the heme-binding domain of Homo CYP51, which is the catalytic site and the target of drug action. (B) NT-a9 is relatively non-toxic to human cells. The CCK8 assay of drugs (NT-a9 or FLC) to human cells (HUVEC or 293T).
